# Anemoside B4 Protects Rat Kidney from Adenine-Induced Injury by Attenuating Inflammation and Fibrosis and Enhancing Podocin and Nephrin Expression

**DOI:** 10.1155/2019/8031039

**Published:** 2019-05-02

**Authors:** Qin Gong, Lu-Ling He, Mu-Lan Wang, Hui Ouyang, Hong-Wei Gao, Yu-Lin Feng, Shi-Lin Yang, Li-Jun Du, Jun Li, Ying-Ying Luo

**Affiliations:** ^1^Jiangxi University of Traditional Chinese Medicine, Nanchang 330006, China; ^2^State Key Laboratory of Innovative Drugs and Efficient Energy-Saving Pharmaceutical Equipment, Nanchang 330006, China; ^3^College of Pharmacy, Guangxi University of Chinese Medicine, Nanning 530000, China; ^4^School of Life Sciences, Tsinghua University, Beijing 100084, China

## Abstract

Anemoside B4 (B4) isolated from* Radix Pulsatilla* has anti-inflammatory activities in the colon and antitumor effects. However, its role in the prevention and treatment of kidney injury has not been reported. Here, we reported the effects of B4 on chronic kidney injury (CKI) and studied its related mechanism based on an adenine-induced kidney injury model in rats. The results showed that serum BUN (blood urea nitrogen), Crea (creatinine), and urinary proteins increased significantly after oral administration of adenine. Meanwhile, the adenine contents in both renal tissue and urine increased markedly compared with those of normal rats. Moreover, IL-1*β*, IL-6, TNF*α,* and NF*κ*B expression was upregulated in the kidney. Simultaneously, the expression of NLRP3 (the nucleotide-binding and oligomerization domain–like receptor, leucine-rich repeat and pyrin domain–containing 3) in the inflammasome, which consists of Caspase 1, ASC (apoptosis-associated speck*-*like protein containing a caspase recruitment domain), and IL-18, was significantly upregulated. B4 could significantly decrease BUN and Crea; reduce urinary proteins in rats; suppress the expression of IL-6, IL-1*β,* NF*κ*B, NLRP3, Caspase 1, ASC, and IL-18; and increase urinary adenine contents and promote its excretion. In addition, B4 also upregulated the expression of podocin and nephrin, two major podocyte proteins, and reduced the fiber collagen in the renal interstitial, suggesting that B4 could protect the glomerular matrix from adenine injury in addition to its anti-inflammatory effects. The results of this study show new perspective of B4 as a potential drug against adenine-induced renal injury.

## 1. Introduction

*Pulsatilla chinensis* (Bge.) Reg (Pulsatilla) is commonly used in traditional Chinese medicine and has antimicrobial and anti-inflammatory functions [1–4]. Saponins are major ingredients in Pulsatilla [5–8]; for example, anemoside B4 (B4), which is isolated from the radix of Pulsatilla, has a content of 4% [9, 10]. Referring to the pharmacological studies on B4 in the latest five years mainly includes the detection of pharmacokinetics, tissue distribution, excretion by LC-MS /MS method, and biotransformation and metabolic profile of anemoside B4 in rat small and large intestine microflora [11–13]. Relevant studies have shown that B4 has antiviral and immunoregulatory effects* in vitro* and* in viv*; B4 could reduce the incidence and severity of porcine circovirus 2 PCV2-induced immunopathological damage, improving the phenomenon of body temperature elevation, weight loss, anaemia, and internal organ swelling, and affect the immunoglobulin levels and protein absorption [14]. B4 might effectively regulate immune responses via upregulated IL-2 expression levels after endothelial cells were challenged with PRRSV [15]. Moreover, B4 has both anti-inflammatory and anticancer effects [16–21]. It has certain cytotoxicity on the human myelogenous leukemia K562 cell line and its metabolites could exhibit a reduction in cell viability of SMMC-7721. In a preliminary study, we found that B4 could protect the kidney from injury induced by a variety factors in mice. Thus, we speculated on its protective effects against renal damage.

Most kidney injury is caused by multiple causes and progressive deterioration, resulting in high levels of nephron destruction, irreversible damage to the renal parenchyma, and the subsequent deterioration of renal function. There are clinical manifestations of metabolite retention, including water, electrolyte, acid-base balance, and systemic involvement [22]. During this pathological process, there are also various associated inflammatory factors, such as TNF*α* and IL-6 [23–25]. Excessive adenine cannot be excreted from the kidney in a timely manner, which could lead to crystal formation and kidney microinflammation, resulting in severe kidney injury [26]. Therefore, adenine is a common agent for inducing chronic renal injury [27], as it interferes with the body's normal adenine metabolism, leading to metabolic disorders. Xanthine oxidase in corroboration with 2,8-dihydroxyadenine, an adenine metabolite that is very difficult to dissolve in water and is often deposited in the renal tubules, causes renal tubular obstruction, which leads to a significant increase in serum uric acid, creatinine (Crea) and urea nitrogen (BUN) [28].

To explore the pharmacological actions of B4, we evaluated the effects of B4 on rat renal injury induced by overloaded adenine in this work. We observed changes in the renal pathology and elucidated the mechanism associated with the actions of B4.

## 2. Materials and Methods

### 2.1. Animals

Male Wistar rats weighing 160g-180g were purchased from Hunan SJA Laboratory Animal Co., Ltd., Changsha, China. This experiment was completed at the Laboratory of Barrier Environment of the Jiangxi Bencao-Tiangong Technology Co., Ltd. (Nanchang, China). The animals were housed in temperature- and humidity-controlled rooms under a 12 h light/dark cycle and provided with unrestricted amounts of rodent chow and drinkable water. All procedures described were reviewed and approved by the Institutional Animal Care & Use Committee of Jiangxi University of Traditional Chinese Medicine (TCM) and the Animal Welfare & Ethics Committee of Jiangxi University of TCM (approval ID: 17-JunLi-B4). The experimental procedure strictly followed the guidelines of the Experimental Animal Welfare and Ethics of China.

### 2.2. Chemical and Materials

**Figure 1:**
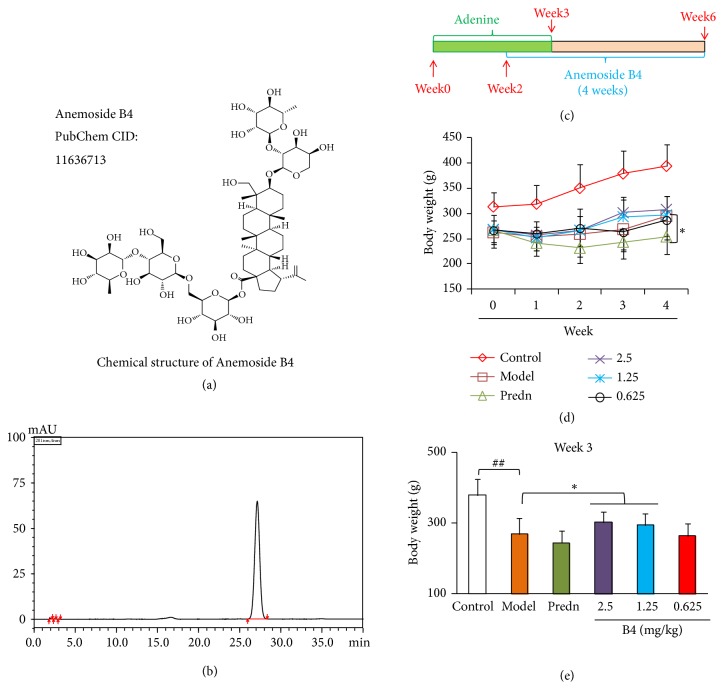
The body weight of rats injured by adenine after the intravenous administration of B4. (a) Chemical structure of B4. (b) The chromatogram of anemoside B4. (c) Time schematic of the experiment. (d) Body weight during 4 weeks. (e) Body weight at week 3. The data were shown as mean ± SEM from 10 rats in each group. ##, compared with the control,* P* < 0.01. *∗*, compared with model groups,* P* < 0.05. Predn: prednisolone, 5mg/kg.

Anemoside B4 (B4), whose chemical structure is shown in Figure 1(a) (Batch No. 20161107, purity of 98% using HPLC determination, the chromatogram of anemoside B4 is shown in Figure 1(b)) was presented by Professor Yu-Lin Feng from the Phytochemical Department of our university. Prednisolone (pred.) (11-beta,17-alpha; 11,17,21-trihydroxypregna-1,4-diene-3,20-dione; (11beta)-11,17-dihydroxy- 3,20- dioxopregna-1,4-dien-21-yl acetate) (Batch No. 161165) was purchased from Xianju Pharmaceutical Ltd. (Zhejiang, China). Adenine (Sigma-Aldrich, Lot#WXBB0585V) was purchased from Sigma (Shanghai, China). Creatinine (Crea), urea nitrogen (BUN), and total protein (TP) kits were purchased from Heguan Chemical Company (Beijing, China). The Bradford Protein Assay Kit (P0006) was purchased from Beyotime Biotechnology Company (Beijing, China). The qPCR detection kit (Lot# L20509) was purchased from Quanshijin BioChem Tech Ltd. (Beijing, China). Hematoxylin (MHS16-500ML, LOT#SLBK4909V) and Eosin Y (HT110116-500ML, LOT#J6425V) were purchased from Sigma (Shanghai, China). Masson's trichrome kits (Lot:0606A18) were purchased from Leagene Biotechnology Co., Ltd. (Beijing, China). A Bio-Rad electrophoresis unit and Bio-Rad ChemiDocXRS+ Gel Imaging System (Beijing, China), LEICA RM2235 paraffin slicer, and LEICA DM2500 Optical Microscope (Beijing, China) were used in this study.

### 2.3. Dosages and Groups

All rats were randomly separated into the following six groups (each group consisting of 10 rats, n=10): the normal control group, model control group, positive control groups, B4 large dose group (2.5 mg/kg), B4 medium dose group (1.25 mg/kg), and B4 small dose group (0.625 mg/kg). All the B4 dosages were determined by the preexperiment, and B4 was given by intravenous administration. Prednisolone, a type of glucocorticoid, was used as positive control (5mg/kg) by oral administration.

### 2.4. Experimental Process

The chronic kidney disease model was referenced in the Ali and Diwan reports [29, 30]. The rat model was created by oral administration of adenine (mixture with 0.5% CMC-Na solution, 220 mg/kg) for 3 weeks. At day 14 after adenine administration, three dosages of B4 were given by intravenous injection continuously for 4 weeks (one time per day) (the schematic protocol is shown in Figure 1(c)). The normal control was given vehicle with 0.5% CMC-Na solution, the model control was given normal saline, and the positive control was given prednisolone. After 4 weeks of administration, the blood and urine samples were collected, and the rats were killed with anesthesia. The rat kidneys were isolated; the right kidney was fixed with 10% formalin and left kidney was stored at -80°C for protein expression analysis.

During the experiment, the general manifestations of the rats, such as their spirit, motor action, and rat fire light and color, were observed and recorded. Additionally, their body weights were detected and recorded each week. Rat urine was collected each week using metabolite cages. The quantity of urine collected in 24 h was determined, and the urine protein content was tested. Serum was isolated from the rat blood for the biochemical tests. The serum BUN, Crea, and TP were tested using a 7100 automatic biochemical analyzer (Hitachi, Japan). The kidney tissue treated with 10% formalin was analyzed by H.E. preparation or by staining the tissue with picrosirius red and Masson's trichrome and making observations with a microscope. Grayscale scanning of the images was carried out by using Adobe PhotoshopCS3 (Adobe, California, USA).

### 2.5. Protein Expression

Protein expression was analyzed using Western blotting as previously described [31, 32]. For Western blot analysis, primary antibodies against NF*κ*B (rabbit polyclonal antibody, ab16502), TNF*α* (rabbit polyclonal antibody, ab6671), NLRP3 (rabbit polyclonal antibody, ab210491), IL-1*β* (rabbit polyclonal antibody, ab9722), IL-6 (mouse monoclonal antibody, ab9324), Caspase 1 (rabbit polyclonal antibody, ab1872), and nephrin (rabbit monoclonal antibody, ab216341) were purchased from Abcam (Shanghai, China). TLR4 (mouse monoclonal antibody, sc-293072) and ASC (mouse monoclonal antibody, sc-271054) antibodies were purchased from Santa Cruz (Beijing, China). The IL-18 (rabbit polyclonal antibody, TA324190) and podocin (rabbit polyclonal antibody, TA351459) antibodies were purchased from ORIGENE (Shanghai, China). The goat anti-mouse IgG-HRP (ZB2305) and goat anti-rabbit (ZB2301) IgG-HRP secondary antibodies were purchased from ZSGB-Bio (Shanghai, China). The targeted proteins were visualized with the Super Signal West Femto Chemiluminescent Substrate (Thermo Scientific Pierce, Beijing, China), and the intensities of the visualized bands were analyzed using the Quantity One software (Bio-Rad, Shanghai, China). *β*-actin (mouse monoclonal antibody, TA-09, Zhongshan Jinqiao Biotech company, Beijing, China) was used as an internal control. The data were expressed as the ratio to *β*-actin.

### 2.6. Detection of Adenine Using LC/MS

Quantitative determination was performed using a Rapid Resolution Liquid Chromatography System (1290 Infinity, Agilent) coupled to a triple quadrupole mass spectrometer (6460, Agilent) [33]. The liquid chromatography system was equipped with a binary pump, a thermostatic column compartment, an autosampler, and an ultraviolet diode-array detector.

Chromatographic separation was achieved on a Welch Ultimate Luna 3*μ*m HILIC 200A (2.0×150mm). The mobile phase was a mixture of 0.1% formic acid in water (A) and acetonitrile (B). The gradient elution was programmed as follows: 95% B,0.01-2 min; 95-80% B,2-3 min; 80-70% B,3-4.5 min; 60-95 % B,5.5- 6 min; and 95% B,6.0-7.0 min. The total run time was 7 min. The column temperature was maintained at 30°C and the injection volume was 2*μ*L. The flow rate was set at 0.25 mL/min. The electrospray ionization (ESI) was performed in positive mode. The mass spectrometer parameters were optimized as follows: drying gas flow rate, 9 L/min; drying gas temperature, 325°C; nebulizer pressure, 45 psi; and capillary voltage, 3000 V. Nitrogen was used in all cases. The precursor product ion pairs used in MRM mode were m/z 136.1-119.5 for epinephrine. The fragmentor was set at 100 V and the collision energy (CE) was set at 23 eV. The dwell time of each ion pair was 100 ms. Instrument control, data acquisition, and evaluation were performed with the Mass Hunter workstation software (Version B.04.00).

### 2.7. Data Analysis

The data were expressed as the mean ± SEM and statistically analyzed using one-way ANOVA analysis, and the* t*-t test was performed for two groups. The testing was performed using the SPSS 19.0 software (IBM, Chicago, USA).* P *values less than 0.05 were considered statistically significant. The statistical graphs were produced using the GraphPad Prism 5.0 software (GraphPad Company, San Diego, California, USA) or the Microsoft Office Excel 2010 software (Microsoft, Maryland, USA).

## 3. Results

### 3.1. Effect of B4 on the General Conditions and Body Weights of the Rats with Adenine-Induced Renal Injuries

After adenine administration, rats in each group showed signs of apathy, coarse and messy fur, weight loss, and decreased activity. After B4 intervention, these phenomena were improved. The rats' body weights are displayed in Figures 1(d) and 1(e). Their weights were obviously reduced, implying that the adenine model could influence body weight. B4 at the large and medium doses could reverse the decrease in weight. The small B4 dose had no effect on weight. Prednisolone could not inhibit the decrease in body weight.

### 3.2. Effect of B4 on Serum Biochemical Indexes in Rats with Adenine-Induced Renal Damage

**Figure 2:**
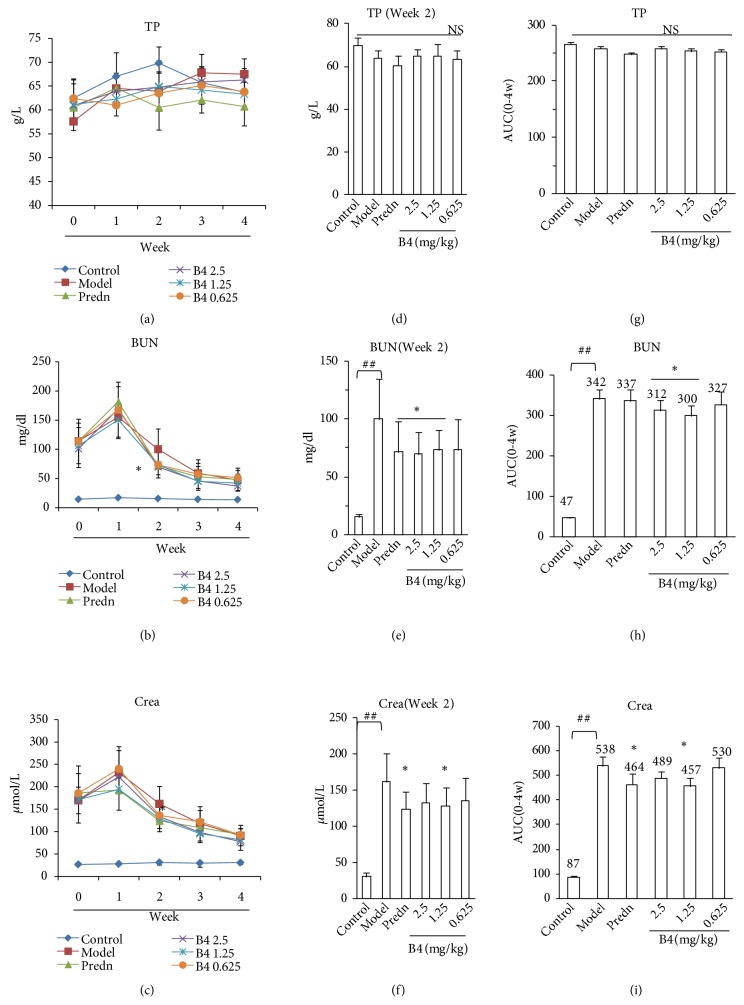
The index of kidney function (BUN and Crea) injured by adenine after the intravenous administration of B4. (a)–(c) Changes of kidney function. (a) Total protein (TP). (b) BUN. (c) Crea. (d)–(e) Kidney function at week 2. (g)–(i) AUCs (area under the curve) of TP, BUN, and Crea during the course. The data were shown as mean ± SEM from 10 rats in each group. ##, compared with the control,* P* < 0.01. *∗*  & *∗∗*, compared with model groups,* P* < 0.05 and* P* < 0.01. Predn: prednisolone.

Figure 2 presents the rats' kidney functions after the intravenous administration of B4. First, adenine could injure the rat kidney, causing a distinct increase in BUN and Crea. B4 at the large and medium doses could suppress the increase in BUN and Crea compared with the model groups after 2 weeks of B4 administration. Based on Figures 2(a), 2(b), and 2(c), we found that BUN and Crea could decrease with time, reflecting kidney self-recovery. The AUCs (area under the curve) of BUN and Crea were calculated to evaluate the effect of B4 on BUN and Crea, respectively. The AUCs of BUN and Crea in the rat model increased compared with those of the control groups, indicating that adenine actually injured the rat kidneys. B4 at the large and medium doses could also decrease the BUN and Crea AUCs, which is in agreement with the BUN and Crea levels at week 2 (Figures 2(h) and 2(i)). The small B4 dose had no effects. Prednisolone could decrease BUN and Crea similar to B4. However, during all the experiments, the blood total protein (TP) displayed negative changes compared with the control groups (Figures 2(a), 2(d), and 2(g)).

### 3.3. Effects of B4 on 24 h Urinary Protein Excretion in Rats within Adenine-Induced Renal Injury

**Figure 3:**
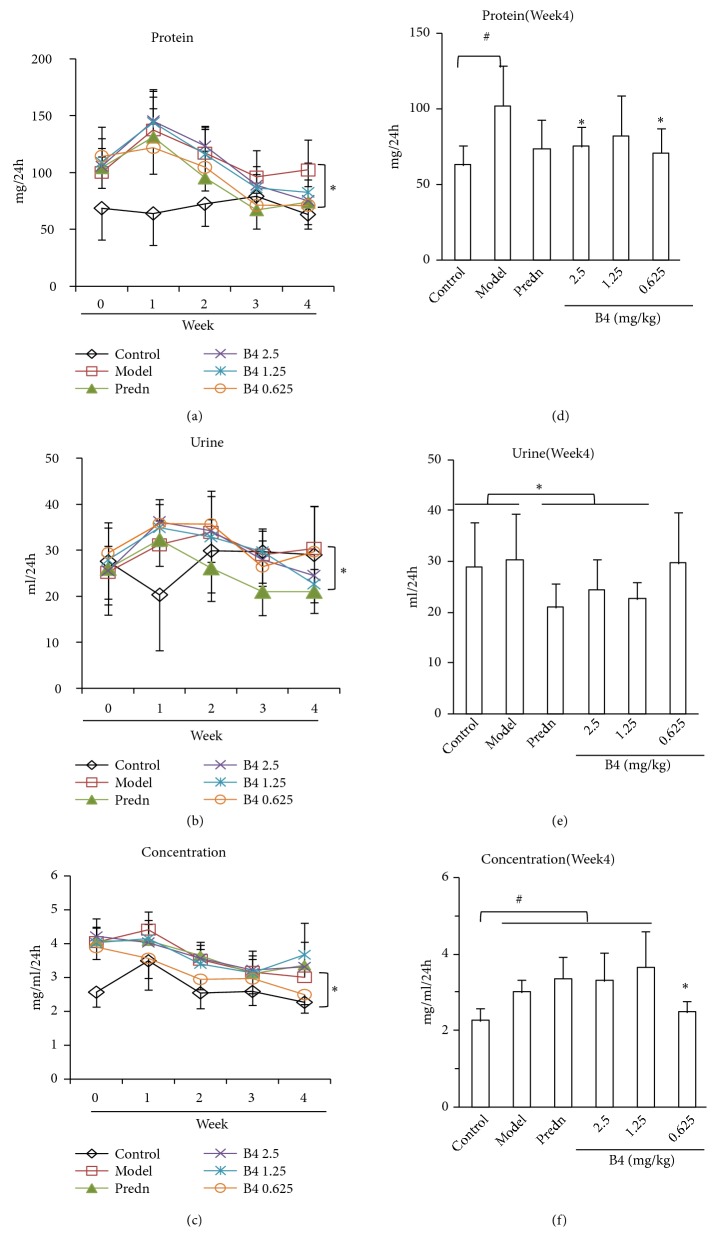
Urine and total proteins in urine of rats after the intravenous administration of B4. (a) Total protein of urine in 24h. (b) Quantity of urine during 24h. (c) Concentration of urinary protein in 24h. (d)–(f) Urinary protein of rats after administration of B4 at week 4. At the end of experiment (week 4), the urinary protein per 24h of the model groups was higher than that of normal rats (control). B4 in large and small dosage decreased the protein content. The data were shown as mean ± SEM from 10 rats in each group. #, compared with the control,* P* < 0.05. *∗*, compared with model groups,* P* < 0.05. Predn: prednisolone.

Urinary protein is a key index reflecting kidney injury. After adenine overload, the urinary protein levels in the model group were higher than those in the control group, and the total urinary protein at 24 h after 4 weeks was also higher than that of the control group (Figure 3). B4 at the large and small doses could reduce the 24h total protein levels, implying that B4 protects the kidney from adenine injury (Figure 3(d)). After 4 weeks of B4 treatment, there was more urine from the model group at 24 h, and there were no differences in the protein concentrations in model groups; however, the model groups were higher than the normal groups (Figures 3(e) and 3(f)). Finally, the total protein in the B4-treated rat was lower than in the model rat, showing that B4 affects kidney damage by decreasing urinary protein levels.

### 3.4. Effects of B4 on the Renal Histopathology of Rats with Adenine-Induced Renal Injury

**Figure 4:**
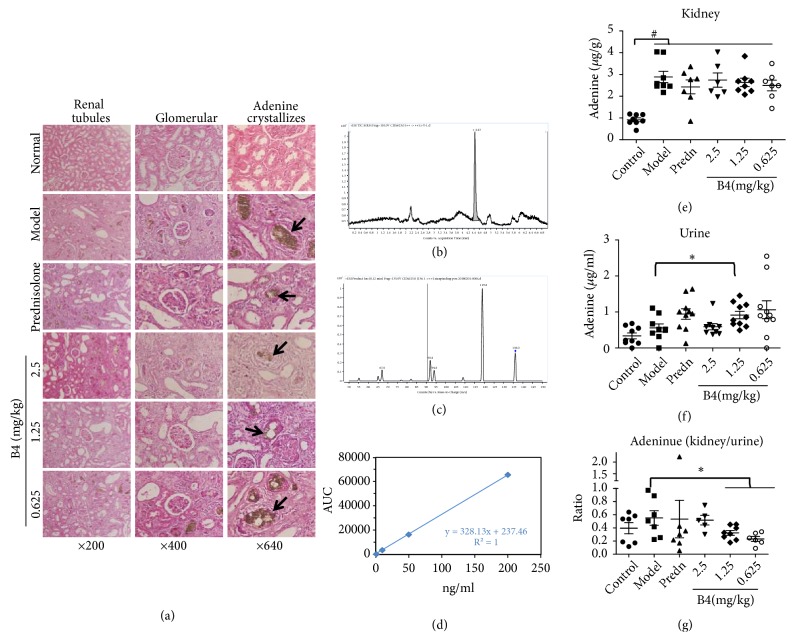
Pathological changes of the rat kidney induced by adenine after the intravenous administration of B4. (a) Morphology of the kidney. The black arrows showed the adenine crystallizes. (b) MRM of B4. The retention time of B4 was at 4.43min. (c) Mass spectrum (MS/MS) of B4. The* m/z* was 136. (d) The standard curve of B4. (e)-(f) Content of adenine in the kidney (e) and urine (f). (g) Ratio of the adenine in kidney to the adenine in urine. The data were shown as mean ± SEM from 7-10 rats in each group. #, compared with the control,* P* < 0.05. *∗*, compared with model groups,* P* < 0.05.

With respect to morphology, the kidney structure of the normal group rats was clear and showed normal glomeruli (renal body), renal tubules, and interstitium. In contrast, the number of nephrons in the model control group decreased, and granulomas were present in the renal interstitium (mainly consisting of macrophage-like lesions). Visible brown adenine crystals were observed in the lumen and interstitium of the renal tubules, and the renal tubules expanded in a reticular pattern and partially disappeared, indicating that adenine could damage the kidney renal tubules (Figure 4). The renal tubule dilation was alleviated in the B4 high- and low-dose groups.

In the model groups, adenine crystallized, implying that the kidney could not handle the adenine overload, and resulted in injury to the kidney tubules. B4 could protect the kidney from the damage caused by the adenine by reducing the number of adenine crystals (Figure 4(a)). To determine the exact adenine contents in the kidney, LC/MS was employed for adenine detection (Figures 4(b)–4(d)). A standard curve was constructed, and the following correlation equation was determined to calculate the adenine concentration: y=326.13x+237.46 (R^2^=1). After adenine overload, the concentration of adenine in the rat kidney was distinctly higher than in the normal control rat. B4 at the small dose and prednisolone could decrease the adenine levels, but the results were not statistically significant (Figure 4(e)). Interestingly, the adenine contents in rat urine from the groups treated with the middle and small B4 doses were higher than those from the model groups, implying that B4 could promote the secretion of adenine in the urine (Figure 4(f)). To confirm this result, we examined the ratio of adenine in the kidney to adenine in urine (adenine in kidney/adenine in urine). In accordance with the adenine contents, this ratio was lower in the middle and small B4 dose groups than in the model groups, and the results were statistically significant, showing that there were higher adenine levels in urine from the B4 groups than in the model groups (Figure 4(g)). The positive control prednisolone displayed increased adenine levels in urine, but the results were not statistically significant.

### 3.5. Effect of B4 on the Expression of Inflammatory-Related Factors in the Renal Tissue

**Figure 5:**
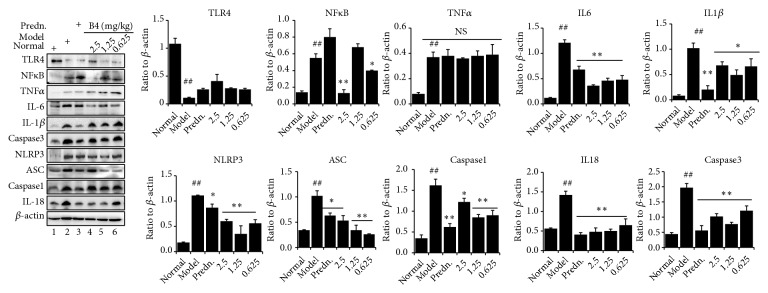
Protein expression of inflammation cytokines after the intravenous administration of B4. The data were shown as mean ± SEM from 10 rats in each group. ##, compared with the control,* P* < 0.01. *∗* and *∗∗*, compared with model groups,* P* < 0.05 and* P* < 0.01. Predn: prednisolone. NS means no significance.

In Figure 5, we found that, in addition to adenine overload, the inflammatory cytokines were dramatically upregulated compared with normal control rats, except TLR4. B4 could suppress the expression of IL-6, IL-1*β, *and NF*κ*B but had a negative effect on TNF*α* expression, indicating that the anti-inflammatory effects of B4 on adenine-induced kidney damage are related to IL-6 and IL-1*β*/NF*κ*B signaling. A recent study showed that adenine crystals stored in the tubules caused localized inflammasome expression, which resulted in kidney damage [24]. In our experiments, the expression of NLRP3 and its components were distinctly upregulated by chronic adenine stimulation. However, B4 could effectively suppress this expression, suggesting that the NLRP3 inflammasome is a major inflammatory pathway after adenine overload. The positive control prednisolone could also attenuate the expression of the NLRP3 inflammasome.

### 3.6. Effects of B4 on Renal Fiber Collagen

**Figure 6:**
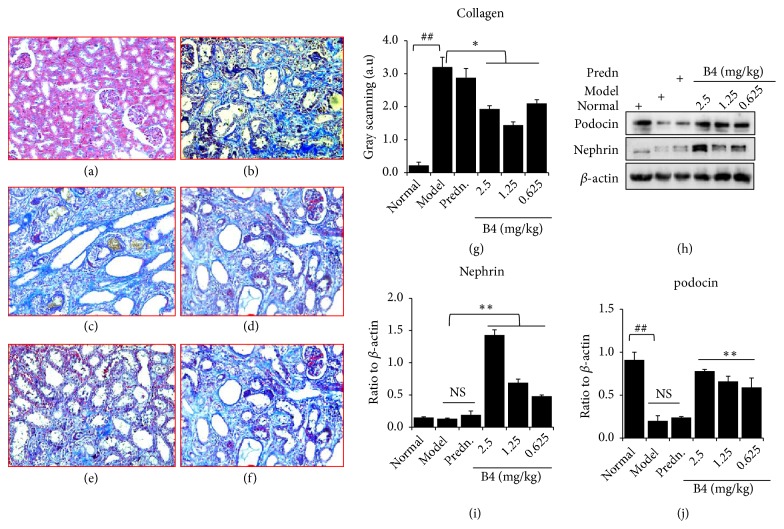
Fibrosis of kidney and expressions of the factors after the intravenous administration of B4. (a)–(f) Morphology of kidney (200×amplification). (a) normal; (b) model; (c) prednisolone (Predn.); (d) B4 (2.5 mg/kg); (e) B4 (1.25 mg/kg); (f) B4 (0.625 mg/kg); (g) the statistical analysis of the fibrosis in the kidney images by using grayscale scanning. (h)-(j) Protein expression of nephrin and podocin. The data were shown as mean ± SEM from 10 rats in each group. ##, compared with the normal control,* P* < 0.01. *∗*, *∗∗*, compared with model groups,* P* < 0.05 &* P* < 0.01. NS means no significance.

Figures 6(a)–6(g) show the fibrous status of the kidney tissue.  Figure 6(a) shows that renal tubulointerstitial fibrosis was less likely in kidneys from normal rats. After adenine overloaded, the rat kidney tissue fibers proliferated significantly. B4 showed a specific inhibition of renal fibrosis, which was evident at the medium dose (Figure 6(e)). The positive control prednisolone showed the ability to promote tubulointerstitial fibrosis (Figure 6(c)), which will require further study. The analytic results by using grayscale scanning present that B4 could attenuate the collagen in the renal tubulointerstitial being consistent with the histological images (Figure 6(g)). The initiation of renal fibrosis is generally thought to be associated with high TGF-*β* (transforming growth factor-beta) expression [34, 35]; therefore TGF-*β*1 might play a role in the effect of B4 on suppress renal tubulointerstitial fibers caused by adenine overloaded, which needs to be studied further. Nephrin and podocin are the main factors in podocyte-associated protein expression, and their expression directly affects the permeability of the glomerular matrix membrane, which in turn affects kidney function [36]. B4 may significantly increase the expression of nephrin and podocin in a dose-dependent manner, indicating that the ability of B4 to prevent and treat renal injury is related to the promotion of nephrin and podocin expression and in turn improving glomerular podocytes (Figures 6(h)–6(j)).

## 4. Discussion

In this study, we observed the pharmacological effects of B4. The results showed that B4 can effectively reduce the increase in BUN and Crea induced by adenine and simultaneously reduce urinary protein levels caused by kidney damage, which are related to the inhibition of inflammatory reactions. Additionally, B4 can also improve renal podocytes by enhancing the expression of podocin and nephrin. It is also interesting that B4 promotes the excretion of the overloaded adenine from the kidney, thereby reducing the renal damage.

Studies have shown that localized microinflammation (microlocation) is common in patients with chronic renal failure (CRF); this further promotes the deterioration associated with kidney disease and the emergence of various complications [37]. The degree of microinflammation has been considered as a reliable indicator of CRF prognosis [38]. Therefore, inflammatory factors, such as IL-6, IL-1*β*, and TNF*α*, should be examined when studying renal function impairment [28]. The expression of the NLRP3 inflammasome should also be examined because it is correlated with chronic inflammation. Our results show that renal inflammatory factor expression was significantly increased in adenine-induced rat kidney injury. B4 could significantly inhibit the expression of IL-6, TNF*α*, and NF*κ*B through this related signaling pathway. However, there were no obvious inhibitory effects on IL-1*β*, which requires further study. Meanwhile, adenine could increase the expression of the NLRP3 inflammasome. In addition to the NLRP3 inflammasome, B4 also significantly inhibited the expression of other three proteins, Caspase 1, ASC, and IL-18, indicating that B4 has a distinct effect on the pathways related to these two kinds of inflammatory responses.

Based on the morphological observations, adenine mainly damages the renal tubules and causes less glomerular damage. In the model group, renal interstitial hyperplasia and tubular dilatation were observed in the kidneys, and adenine crystals were observed in the renal tubules. Inflammatory hyperplasia could be observed around the crystalline tubules, and the wall of the tube was thickened. B4 could improve this kind of tubular hyperplasia to a certain extent and reduce crystallization. The results of the adenine test in the kidney tissue and its excreted urine revealed that B4 could increase adenine levels in rat urine, suggesting that B4 can promote adenine excretion. Excessive adenine excretion from the body could reduce not only the accumulation of crystals in the renal tubules but also the renal tubular damage and inflammatory reactions caused by these crystals.

It is generally believed that renal damage caused by adenine occurs mainly in the renal tubules [39]. Our results showed that urinary protein levels increased significantly in the model group and that B4 could significantly reduce urinary protein levels. Because the increase in urinary protein levels is mainly due to glomerular basement membrane damage, it is suggested that adenine could also damage the glomerular matrix and that B4 could not only act on the renal tubules but also protect the glomerulus from injury.

Glucocorticoids are commonly used drugs in the treatment of acute and chronic kidney injury [40]. Therefore, prednisolone, an active glucocorticoid metabolite, was employed as a positive control in this study [41]. The results showed that prednisolone had a specific effect on adenine-induced renal injury by inhibiting the expression of inflammatory factors and improving renal function. Prednisolone reduced urinary protein levels but had no effect on urinary adenine excretion. Additionally, prednisolone did not improve body weight loss during adenine-induced kidney damage, while B4 could increase rat body weights during adenine overload. In particular, the increase in body weight was significant and statistically significant compared with the model group at the large and medium B4 doses. In addition to its anti-inflammatory role, B4 could also reduce urinary protein levels and accelerate the excretion of adenine.

During this pathological process, podocin and nephrin were significantly downregulated in the model group, while B4 could significantly upregulate their expression, indicating that the protective effects of B4 in the kidney involve protection of the glomerular basement membrane and maintenance of normal podocyte physiological state. Prednisolone had no significant effects on podocin and nephrin expression, implying that the role of this hormone may be mainly to inhibit the expression of inflammatory factors but not to improve the function of the basement membrane, which may be one of the most important differences between prednisolone and B4. B4 can not only inhibit the expression of inflammatory factors but also upregulate the expression of nephrin and podocin and the podocyte function-related proteins, thereby protecting the glomerular basement membrane from damage induced by adenine.

Adenine causes renal tubulointerstitial fibrosis [27, 42, 43], which was also confirmed in our experiments. Our results showed that there was significant fibrosis in the renal tubulointerstitial 6 weeks after adenine overloaded. B4 can inhibit renal interstitial fibrosis distinctly. The mechanism for the B4 inhibition of renal fibrosis caused by adenine remains to be further studied.

Taken together, B4 may be effective for treating chronic kidney injury (CKI) induced by adenine. The mechanism of B4 in CKI is correlated with decreasing BUN, Crea, and urinary protein levels; protecting glomeruli; promoting adenine excretion; and inhibiting the expression of inflammatory factors, thereby attenuating renal tubular injury. Additionally, B4 can also upregulate the expression of nephrin and podocin, preventing the glomeruli from injury induced by adenine.

## Data Availability

The data used to support the findings of this study are included within the article.
